# Predictors of community versus specialty mental health service use: a prospective cohort study

**DOI:** 10.1007/s00787-020-01575-8

**Published:** 2020-06-18

**Authors:** Maria Larsen Brattfjell, Thomas Jozefiak, Lars Wichstrøm

**Affiliations:** 1 Department of Psychology, Norwegian University of Science and Technology, 7491 Trondheim, Norway; 2 Faculty of Medicine and Health Sciences, Institute of Mental Health RKBU, Norwegian University of Science and Technology, P. O. Box 8905, 7491 Trondheim, Norway; 3 Department of Child and Adolescent Psychiatry, St. Olavs Hospital, Torgarden, P. O. Box 3250, 7006 Trondheim, Norway

**Keywords:** Service use, Community services, Child and adolescent mental health specialized services, Children, Mental health problems

## Abstract

Child and adolescent mental health specialized services (CAMHS) are supposed to serve those who are most seriously disturbed and impaired. However, little is known about how children receiving treatment at different levels of care differ. The present study seeks to determine whether having a psychiatric disorder and resulting impairment measured in early childhood increases the odds of receiving help in CAMHS versus from community services during middle childhood or whether other factors (e.g., parenting stress, family functioning) also influence service utilization. A screen-stratified sample (*n* = 995 of the 2003–2004 birth cohorts) in Trondheim, Norway was assessed biennially from age 4–12 with semi-structured diagnostic interviews and recording of service use, family functioning, parental perceived need, and parenting stress. Behavioral disorders more strongly predicted CAMHS than community service use, whereas impairment predicted community service use. However, impairment increased the odds of receiving services in CAMHS if the parents perceived a need for help. Parental perceived need for help also increased the odds of CAMHS use independent of diagnosis and impairment. Having an emotional disorder, attention deficit/hyperactivity disorder (ADHD), parenting stress, previous service use, or family functioning did not predict service use at either level. Whereas children with behavioral disorders received help from CAMHS, children with emotional disorders did not receive services at either level. ADHD did not predict service use, indicating that young children with ADHD without comorbid disorders are not sufficiently detected. Efforts to detect, refer and treat emotional disorders and ADHD at the appropriate level should be increased.

## Introduction

Psychiatric disorders in children are prevalent, with a current global estimated prevalence of 13% [1]. However, many children with psychiatric disorders do not receive treatment [2–4]. Mental health services are costly, but the consequences of not receiving help are even larger [5]. Most countries divide services to children into community services and specialized child and adolescent mental health services (CAMHS). The latter typically handles the most serious, complicated and debilitating conditions, whereas community services take on milder cases. This division of labor intends to optimize treatment in a cost-efficient way [6–9]. However, if those in need of CAMHS are not referred to such services, optimal treatment may not be delivered, and conversely, if children with less serious conditions are treated in CAMHS, costly treatment may be offered to children who could be effectively treated with less extensive interventions. Yet few studies have explored how children treated in community services and CAMHS differ.

The main foci considered in most national guidelines for intake to CAMHS are problem severity and expected gain. It is assumed that patients receiving treatment in CAMHS will fulfill diagnostic criteria and be admitted based on characteristics of their mental health problems (i.e., severity, diagnosis, and impairment) [6–9]. However, the results of studies exploring these factors as predictors of children’s use of CAMHS have been inconsistent [10–13]. While these studies have demonstrated that external factors, such as parental burden and ethnicity, also influence service use, none have examined whether predictors of CAMHS and community services differ. The Method section provides more information about these services in Norway; the country where the present study was conducted.

Given current guidelines, we expect that children with a psychiatric disorder as opposed to subclinical conditions are more often referred to CAMHS. However, the community level can—and is expected to—manage many emotional disorders (e.g., simple phobias, non-debilitating depression) [6]; hence, a distinction is made between emotional and behavioral disorders. Unlike other behavioral disorders, attention deficit/hyperactivity disorder (ADHD) is currently—for the most part—considered a neurodevelopmental disorder. Furthermore, ADHD assessment and diagnostic evaluation should be carried out in CAMHS in Norway. Thus, separating ADHD from other behavioral disorders was considered appropriate. Severe impairment in everyday functioning is stated as a reason for referral to CAMHS by several guidelines [6–8]. Thus, we expect persistent impairment to predict use of CAMHS over community services. Not surprisingly, previous service use predicts future service use [14], and the propensity for continued use or re-referrals could differ as re-entry to services might be quicker for previously treated children. Furthermore, assessments in community services may reveal more serious pathology, and when community interventions do not succeed, further referral to CAMHS could be warranted [9].

Failing to manage a child’s problems might impair family functioning and increase parenting stress. To ease such problems, parents might request and obtain services. Lavigne et al. [15] found that family conflict increased use of primary care among children with psychiatric disorders. They hypothesized that families with increased family conflicts might have greater difficulty in implementing treatment recommendations. In another study [16], poor family functioning was found to be associated with further referral to CAMHS. Based on these findings, it is possible that family factors such as poor family functioning and parenting stress might affect the effectiveness of treatment in primary care, and increase the probability of further referral to CAMHS. Furthermore, parents are often the ones instigating a referral-seeking process. Therefore, children of parents who perceive a need could be referred more often than children of parents who do not perceive need for help.

The present study seeks to determine whether prespecified criteria (i.e., having ADHD, an emotional or behavioral disorder, impairment from such disorders, previous service use, parenting stress, family functioning, and parental perceived need for help) measured in early childhood increase the odds of receiving help in CAMHS or community services during middle childhood. We also hypothesize that the effects of family factors and having a psychiatric disorder will be mediated through increased impairment and parental perceived need.

## Materials and methods

### Recruitment and participants

Data are drawn from five time points from the Trondheim Early Secure Study [17], a longitudinal cohort study which followed a community sample of children from the 2003 and 2004 birth cohorts with biennial examinations from the age of 4 in the city of Trondheim (~195,000 inhabitants), Norway. All children and parents attending the ordinary health checkup for 4-year-olds at their local well-child clinic were invited. In total, parents of 3456 children received a letter inviting them to participate in the study, and 3358 attended the well-child clinic. Parents with insufficient proficiency in Norwegian language were excluded (*n* = 176) and 166 parents were missed being asked to participate. Among the 3016 approached, 2475 consented (82%). Based on stratification detailed below, 1250 were drawn to participate further.

The strength and difficulties questionnaire (SDQ P4-16) [18], a screening tool for emotional and behavioral problems was sent to the children’s homes along with the invitation letter. The total difficulties score, ranging from 0 to 40 with a higher score reflecting more problems, were used to generate four strata (cut offs: 0–4, 5–8, 9–11, 12–40). Participants from the strata with higher SDQ-scores were oversampled to increase variability and inclusion of those with the highest likelihood of service use (drawing probabilities of 0.37, 0.48, 0.70, and 0.89, respectively). We succeeded in obtaining diagnostic and service use information from 995 of 1250 consenting participants (79.6%, *M*_age_ = 4.5, SD = 0.25). Follow-ups were performed after two (*T*2, *M*_age_ = 6.7, SD = 0.19, *n* = 752), four (*T*3, *M*_age_ = 8.8, SD = 0.24, *n* = 670), six (*T*4, *M*_age_ = 10.5, SD = 0.17, *n* = 678), and eight years (*T*5, *M*_age_ = 12.5, SD = 0.67, *n* = 638). Parenting stress (OR = 1.02, CI 1.01–1.02, *P* < 0.001) and lower socioeconomic status (SES; OR = 1.69, CI 1.44–1.99, *P* < 0.001) at *T*1 and *T*2 predicted attrition at follow up.

### Setting and the Norwegian health care system

In Norway, health services for those ≤18 years of age are free of charge. In 2017, CAMHS in the health region in question served 8897 children (5.8% of the population), of whom 8.7% were younger than 7 years old, and 36.6% were between ages of 7 and 12 years old, closely resembling national rates. Outpatient services was provided to 95% of the patients, whereas the remaining 5% received inpatient treatment [19]. There are several CAMHS within each health region, and one CAMHS can serve several municipalities. Community services include well-child clinics, school health services, general practitioners, social services, child protection services, and educational and psychological counselling services. There is no available information concerning the number of users of community services.

### Measures

#### Child and adolescent service assessment (CASA)

Parents were interviewed at *T*1–*T*5 using CASA [20]. CASA defines services as efforts to identify, diagnose, or treat emotional, behavioral or substance-related problems. Very few children had used CAMHS at ages 4 (*n* = 11, 1.1%) and 6 (*n* = 14, 1.4%). Because the intention in the present study was to investigate predictors from early childhood of service use in middle childhood, data from *T*1 and *T*2 were combined to create a predictor variable reflecting service use regardless of type at ages 0–6 (0 = for no service use, 1 = service use at *T*1 or *T*2, ‘2 = use at both *T*1 and *T*2). A similar procedure was used for all other predictor variables. Two outcome variables were created: use of ‘CAMHS’ and ‘Community services’ at ages 7–12 years old.

#### Preschool age psychiatric assessment (PAPA)

The PAPA [21], is a semi-structured psychiatric interview used to determine diagnostic and statistical manual of mental disorders (fourth edition) (DSM-IV) defined symptoms and disorders in preschoolers [22]. Interviewers trained by the team that developed the PAPA interviewed parents at *T*1 and *T*2. Diagnoses are set according to computer algorithms. We divided these into ‘behavioral disorders’ (i.e., oppositional defiant disorder and conduct disorder), ‘emotional disorders’ (i.e., major depression, dysthymia, depression not otherwise specified, separation anxiety disorder, generalized anxiety disorder, social phobia, specific phobia, agoraphobia, selective mutism, and obsessive–compulsive disorder), and ‘ADHD’. Because we were specifically interested in impairment resulting from these disorders, impairment was not included in the diagnostic algorithms but was instead kept separate. Ten percent of the diagnostic interviews were recoded by blinded raters and the interrater reliability between them proved good to very good (*κ* = 0.78–0.96).

#### Impairment and perceived need for help

The PAPA asks for impairments related to reported symptoms in 19 different areas of functioning (e.g., relationship with parents, school functioning, and play) according to the World Health Organization’s International Classification of Functioning, Disability and Health (ICC = 0.83) [23]. Furthermore, parents are asked if they think their child needs professional help for the reported problems (*κ* = 0.80).

#### McMaster family assessment device (FAD)

Parents completed the ‘general functioning scale’ of FAD [24], tapping the overall functioning of the family in areas of problem solving, communication, clarity of roles, affective responses, affective involvement, and behavioral control (i.e., clarity and enforcement of family rules). This subscale consists of 12 questions scored from 1 to 4, with a high score indicating poorer global family functioning. Internal consistency was high (*α* = 0.89–0.90).

#### Parenting stress index (PSI)

The PSI measures perceived stress by a parent in the parenting role in relation to a specific child aged <12 years old [25]. A composite score consisting of subscales in the child domain, including reinforces parents, demandingness, and acceptability, as well as in the parent domain, including competence, isolation, attachment, health, role restriction, and spouse, was used. Reliability analysis of the items constituting the composite score indicated acceptable reliability (*α* = 0.85–0.87).

#### Socioeconomic status (SES)

Information about occupational status was provided by parents and coded based on the International Labour Organization’s scheme for classifying occupations [26], which includes six categories ranging from unskilled workers to leaders. When both parents were present, their occupational statuses were averaged.

### Statistical analyses

Analyses were conducted in Mplus version 8.1 [27], using a robust maximum likelihood estimator, and missing data were handled according to a full information maximum likelihood procedure. First, we examined predictors of use of community services and CAMHS using bivariate logistic regression. Second, we tested a multivariate model consisting of only the hypothesized distal variables as predictors (i.e., emotional disorder, behavioral disorder, ADHD, previous service use, family functioning, and parenting stress). Third, these predictors were regressed on impairment. Finally, we tested an indirect path from distal predictors through impairment and in turn via perceived need for help while adjusting for all direct paths to service use. Differences in the magnitude of the coefficients for community services use and CAMHS were tested by comparing the Akaike information criterion (AIC) [28], in a model in which the coefficients were fixed to be identical for the two outcomes, one at a time, with a model in which the coefficients were freely estimated. Because of oversampling, population weights were applied to arrive at correct population estimates.

## Results

The share of participants that had received services increased substantially from *T*1 to *T*2, but remained at level in the years to follow (Table 1). Children receiving services from CAMHS more commonly fulfilled the DSM-IV criteria for a psychiatric disorder. Table 2 provides descriptive statistics for the predictor variables. Community services were provided at *T*1 (*n* = 44) at the following locales: educational and psychological counseling services (2.3%), private practice (2.3%), child protection services (6.8%), social office (0.0%), or a local well-child clinic (47.7%). The remaining received specialty mental health services (11.4%) or another unclassified form of service (38.6%). At *T*2 (*n* = 137), community services were provided at the following locales: educational and psychological counseling services (10.9%), private practice (12.4%), child protection services (0.7%), social office (0.7%), and a local well-child clinic (21.9%), support center (0.7%) and at home (11.7%). The remaining received specialty mental health services (10.9%) or another unclassified form of service (58.4%). The sum of the percentages exceeds 100% as come children utilized several services.

**Table 1:** Percentage of children using services in relation to mental health problems at *T*1–*T*5.

Service use	*T*1	*T*2	*T*3	*T*4	*T*5
Community services	3.1	6.4	5.6	5.1	4.2
CAMHS	1.1	1.4	1.2	2.5	2.9
Other services	0.2	5.6	4.2	4.8	3.3
Total service use	4.4	13.4	11.0	12.4	10.4
Amount with psychiatric disorder in community services	6.5	9.7	13.2	15.2	9.9
Amount with psychiatric disorder in CAMHS	27.3	50.0	66.7	72.0	41.3

Psychiatric disorders refers to emotional or behavioral disorder according to DSM-IV criteria

**Table 2:** Descriptive statistics for predictor variables included in the analysis

Variables	Mean	SD	Min	Max
Service use at age 4–7	0.09	0.30	0	2
Emotional disorders at age 4–7	0.15	0.39	0	2
Behavioral disorders at age 4–7	0.07	0.26	0	2
ADHD	0.02	0.14	0	2
Impairment	0.15	0.39	0	2
Family functioning	1.65	1.28	1	3.25
Parenting stress	183.40	13.54	117.78	273.90
Parental perceived need for help	0.07	0.26	0	2

Separate logistic regression analyses conducted on gender, SES, and age revealed no significant association with community services or CAMHS use: gender (OR = 1.00, 95% confidence interval (CI) 0.64–1.57, *P* = 0.99; OR = 0.66, CI 0.35–1.24, *P* = 32); SES (OR = 1.11, CI 0.89–1.37, *P* = 0.37; OR = 0.98, CI 0.74–1.28, *P* = 86); and age (OR = 1.00, CI 1.00–1.01, *P* = 0.32; OR = 1.00, CI 1.00–1.00, *P* = 0.81). These potential predictors were not included in further analyses.

Emotional and behavioral disorders were bivariately predictive of CAMHS (model 1; Table 3), whereas impairment, previous service use, parenting stress and perceived need for help predicted both CAMHS and community services. However, there were no significant differences in predictive strength vis-a-vis community services and CAMHS. Second, in a multivariate model, behavioral disorders predicted CAMHS, whereas previous service use predicted both community services and CAMHS (model 2). As in the bivariate model, no significant differences between community services and CAMHS were identified. When impairment was included (model 3), it proved predictive of community services, whereas CAMHS was predicted from prior service use and behavioral disorders.

**Table 3:** Predictors during early childhood of community services and CAMHS use in middle childhood

Model		Predictor variables	Community services			CAMHS		
			Beta	95% CI		Beta	95% CI	
				Lower	Upper		Lower	Upper
Bivariate models								
1	Emotional disorder		0.09	−0.38	0.56	0.57^*^	0.04	1.11
	Behavioral disorder		0.44	−0.13	1.01	1.34^***^	0.70	1.98
	ADHD		0.79	−0.12	1.70	0.75	−0.09	1.60
	Impairment		0.64^**^	0.26	1.01	0.65^*^	0.15	1.15
	Service use		0.75^**^	0.23	1.27	1.02^**^	0.41	1.63
	Family functioning		0.24	−0.41	0.89	−0.13	−0.98	0.73
	Parenting stress		0.01*	0.00	0.02	0.01*	0.00	0.02
	Perceived need for help		0.56*	0.03	1.09	1.33^***^	0.75	1.90
Multivariable models								
2	Emotional disorder		−0.07	−0.54	0.40	0.25	−0.31	0.82
	Behavioral disorder		0.22	−0.34	0.79	1.12^**^	0.32	1.92
	ADHD		0.40	−0.61	1.41	−0.10	−1.03	0.82
	Service use		0.58^*^	0.04	1.12	0.76^*^	0.08	1.45
	Family functioning		−0.32	−1.18	0.53	−0.96	−2.23	0.30
	Parenting stress		0.01	0.00	0.02	0.01	−0.00	0.03
3	Emotional disorder		−0.18	0.67	0.32	0.24	−0.36	0.84
	Behavioral disorder		0.06	−0.55	0.67	1.01^**^	0.34	1.87
	ADHD		0.24	−0.76	1.25	−0.12	−1.04	0.81
	Service use		0.47	−0.07	1.01	0.75^*^	0.05	1.44
	Family functioning		−0.35	−1.22	0.52	−0.96	−2.23	0.30
	Parenting stress		0.01	−0.00	0.03	0.01	−0.00	0.03
	Impairment		0.47^*^	0.03	0.91	0.05	−0.51	0.61
4	Emotional disorder		−0.16	−0.67	0.35	0.13	−0.52	0.79
	Behavioral disorder		0.06	−0.55	0.67	1.12^**^	0.28	1.95
	ADHD		0.26	−0.73	1.25	−0.17	−1.08	0.74
	Service use		0.49	−0.08	1.06	0.60	−0.11	1.32
	Family functioning		−0.34	−1.22	0.54	−0.96	−2.22	0.29
	Parenting stress		0.01	−0.00	0.02	0.01	−0.00	0.03
	Impairment		0.50*	0.03	0.98	−0.32	−1.13	0.48
	Perceived need for help		−0.12	−0.83	0.59	1.00*	0.16	1.84

^*^Significant at *p* < 0.05; ^**^significant at *p* < 0.005; ^***^significant at *p* < 0.001

In model 4, parental perceived need for help was added as a predictor, which predicted CAMHS along with behavioral disorders. Notably, the effects of behavioral disorders and parental perceived need for help were stronger predictors of CAMHS than of community service use, as shown by the lower AICs (ΔAIC = 2.01 and 2.00, respectively) obtained between CAMHS and community services when effects were freely estimated as opposed to fixed to be equal. Impairment predicted the use of community services directly. However, although there was no direct path from impairment to CAMHS, impairment predicted use of CAMHS indirectly through increased parental perceived need for help (indirect *B* = 0.30, CI 0.04–0.57, *P* = 0.02). In part, this indirect effect on CAMHS use originated from behavioral disorders, which predicted incapacity (*B* = 0.32, CI 0.15–0.50, *P* = 0.000), which in turn predicted an increase in parental perceived need for help and then CAMHS use (overall indirect *B* = 0.10, CI 0.01–0.18, *P* = 0.02).

## Discussion

This is the first study to examine how predictors of children’s use of community services and CAMHS for mental health problems differ. Early childhood behavioral disorders, but not emotional disorders or ADHD, predicted the use of CAMHS. Parental perceived need for help increased the odds of use of CAMHS, independent of diagnosis and impairment, and the effect of behavioral disorders on CAMHS use partly worked by increasing parental perceived need of help. Impairment predicted the use of community services directly, and indirectly via increased parental perceived need for help.

### Diagnoses

Behavioral disorders were significant predictors of CAMHS use. Although children with behavioral disorders fulfill the criteria for receiving treatment in CAHMS, community services in Norway offer programs for behavioral disorders, such as parent management training [29]. Our findings indicate that these services are underutilized or that the children in our sample had severe behavioral disorders or comorbid psychiatric disorders warranting referral to CAMHS. In general, comorbidity may influence the referral process due to increased severity and impairment.

Unexpectedly, ADHD did not predict service use at any level. Because there is high comorbidity between ADHD and other behavioral disorders in young children [30], those with ADHD might still obtain help from services. Even so, hyperactivity, impulsivity and attention problems could be perceived as age-appropriate by parents and day care personnel in the preschool years, stalling a referral process. When these children enter school and the problems become more evident due to the greater demands placed on attention and behavioral regulation in school as opposed to day care; referral rates may eventually increase [31].

Emotional disorders are arguably less visible and troublesome for the surroundings than behavioral disorders [14, 31, 32], possibly resulting in less motivation for parents to seek help. Moreover, behavioral problems are more stable than emotional problems [33]. Hence, parents and others may experience these problems wax and wane, resulting in a “wait-and-see” attitude [34, 35]. However, for many children, the emotional problems do persist [36]. Thus, one would still expect emotional disorders, if they were recognized as such, to be a significant predictor of service use. Hence, we should not overlook the possibility that emotional disorders are not detected or labeled otherwise by parents and teachers (e.g., shy, sullen, careful, introverted). Community services function as a gateway to other services [37, 38]. Lack in referrals of children with emotional disorders to CAMHS are justified for milder emotional problems appropriately treated in community services. However, emotional disorders did not predict the use of community services, indicating weaknesses in the detection of children with emotional disorders. Thus, means to increase knowledge among parents and teachers about how such problems manifest in children may be warranted.

### Impairment and parental perceived need for help

Contrary to our expectations, impairment in everyday functioning predicted use of community services but not CAMHS. However, impairment predicted CAMHS indirectly through increased parental perceived need for help. Other studies have suggested that impairment might operate through parental perceived need for help as a predictor of any service use [14, 39]. Our findings suggest that once children evince the impairment, parents tend to perceive a greater need to obtain help than when the impairment is low, and they might thus instigate a process of receiving help from CAMHS rather than from community services. This falls in line with another finding of this study, wherein children whose parents perceive a need for help receive help at a higher level, independent of diagnosis and impairment. The fact that parental perceived need for help increases the probability of receiving treatment (distress and amount of problems adjusted) could mean that some of the most vulnerable children, such as children in child welfare and neglected children whose care providers are arguably less inclined to respond to their impairment, are even less likely to receive help. It is known that child welfare at times has displayed suboptimal cooperation with other services and that this might result in children not receiving appropriate treatment for serious and complex mental health problems [40, 41]. Measures to increase parental awareness of children’s mental health problems (e.g., community based education programs) may increase referrals and thus access to services for children in need.

### Limitations

We acknowledge several limitations. First, a sizeable amount of service use was categorized as ‘other’. This group could include services belonging in the CAMHS or community services. Second, our set of predictors was naturally limited. Other factors, perhaps most notably organization of health care, may also affect service use. Third, it is possible that the parents of the children with the largest impairments were among those who declined the invitation to participate in the study or who elected not to participate from *T*2 onwards. This could mean that the most impaired children, who are more likely to be appropriately treated in CAMHS, were underrepresented in the study. Fourth, attrition between time-points is a potential source of bias that could affect the generalizability of the findings. However, a full information maximum likelihood procedure was applied to minimize the effect of missing data. Finally, diagnoses were based on interviews with one parent. The problems identified could be perceived differently by the other parent or even by the child. Even so, using a diagnostic interview instead of a questionnaire is also one of this study’s notable strengths.

## Conclusion

Many children with psychiatric disorders do not receive treatment. Successful referral of patients to the appropriate level of care is essential to secure the best possible treatment in the most cost-efficient way. Children with behavioral disorders had increased probability of receiving services from CAMHS, whereas children with emotional disorders or ADHD did not receive help from either community services or CAMHS. Increased efforts to detect and refer to young children’s emotional problems and ADHD-like behavior are warranted. Prolonged impairment increased the odds of receiving help from community services. However, children with impairment had increased odds of receiving help from community services if their parents perceived a need for help. To the extent that parental perceived need for help is translated into parental action, our results indicate that advocating and pushing for services pays off, especially with respect to the CAMHS system.
